# Long-Term Health-Related Quality of Life of Surgically Treated Pituitary Adenoma Patients: A Descriptive Study

**DOI:** 10.5402/2012/675310

**Published:** 2012-12-31

**Authors:** A. Raappana, T. Pirilä, T. Ebeling, P. Salmela, H. Sintonen, J. Koivukangas

**Affiliations:** ^1^Department of Otorhinolaryngology-Head & Neck Surgery, Institute of Clinical Medicine, University of Oulu, Oulu, Finland; ^2^Department of Otorhinolaryngology, Lapland Central Hospital, Rovaniemi, Finland; ^3^Institute of Clinical Medicine, Faculty of Medicine, University of Oulu, Oulu, Finland; ^4^Department of Public Health, Hjelt Institute, University of Helsinki, Helsinki, Finland; ^5^Department of Neurosurgery, Institute of Clinical Medicine, University of Oulu, Oulu, Finland

## Abstract

*Context*. The literature concerning the health-related quality of life (HRQoL) of patients with surgically treated PA is controversial. *Objective*. To describe the long-term HRQoL of surgically treated patients in all PA classes. *Design and subjects*. The 15D, a generic HRQoL instrument producing a 15-dimensional profile and a single 15D index score (a difference ≥0.03 on a 0-1 scale is considered clinically important), was used to assess the HRQoL of a 13-year surgical cohort of PA patients in Northern Finland. *Results and Conclusion*. Nighty-eight eligible consecutive patients with surgically treated PA were studied at an average of 6.3 years after their latest pituitary operation. The average postoperative 15D profiles in patients with non-functioning PA and in acromegalics without GH-suppressive medical treatment were similar to those of the age-standardized general population. However, after this rather long followup, the mean 15D score and the number of statistically significant 15D dimension impairments, compared with those of their reference population, were 0.11 and 9/15, 0.10 and 3/15, and 0.08 and 7/15 for Cushing's disease, acromegalics needing somatostatin analog, and prolactinoma patients, respectively. Hypopituitarism with replacement medication was not associated with impaired HRQoL. The somatostatin-analog-associated HRQoL finding warrants further clinical research.

## 1. Introduction

Pituitary adenomas (PAs) are benign neoplasms of the adenohypophysis, with an overall annual incidence of about 4/100 000. Prolactin-secreting prolactinomas and clinically non-functioning (NF) PAs are the most common PA classes, followed by, with decreasing incidence, GH- (acromegaly), ACTH- (Cushing's disease, CD), and TSH-secreting (thyrotropinoma) adenomas [1]. The treatment objectives in patients with pituitary adenoma are to eliminate possible mass effects of the tumor, to normalize possible hormonal and metabolic disturbances of the patient, and to recover normal health-related quality of life (HRQoL). Despite the endocrinologically and radiologically successful treatment of a hormonally active or non-functioning PA, both physical and mental adverse effects of the disease may persist thus causing permanent impairment of HRQoL [2, 3]. In the literature controversies exist about correlation of metabolic markers and subjective well-being of the patient, especially in acromegaly [4–8]. Also, there are controversies as to which PAs are associated with the most prominent HRQoL impairments [9, 10], and whether treated NFPAs cause HRQoL impairment at all [11, 12].

HRQoL assessment is important in clinical practice, as it gives the clinician information on issues that have paramount importance for the patients' everyday life. In addition, it has been proposed that evaluation of HRQoL should be combined with hormonal assessment to detect more sensitively patients' improvement during augmented medical therapy in acromegaly [4, 8]. Taking into account these multiple controversies and the emerged importance of patients' subjective morbidity, our objective in this study was to use a generic, validated HRQoL instrument with domestic age-adjusted reference populations in a 13-year cohort of surgically treated PA patients in Northern Finland to evaluate multiple factors influencing the long-term HRQoL of these patients.

## 2. Patients and Methods

In this study we included all surgically treated PA patients found in the PA incidence study in Northern Finland [1] between the years 1992 and 2004. The 15D questionnaire was sent to all patients, and nonrespondents were contacted a second time. In the same inquiry letter, the patients were asked to state their three most significant symptoms and the severity of these symptoms (on a four-step scale), which they associated with their PA before treatment and at the present time.

**Figure 1:**
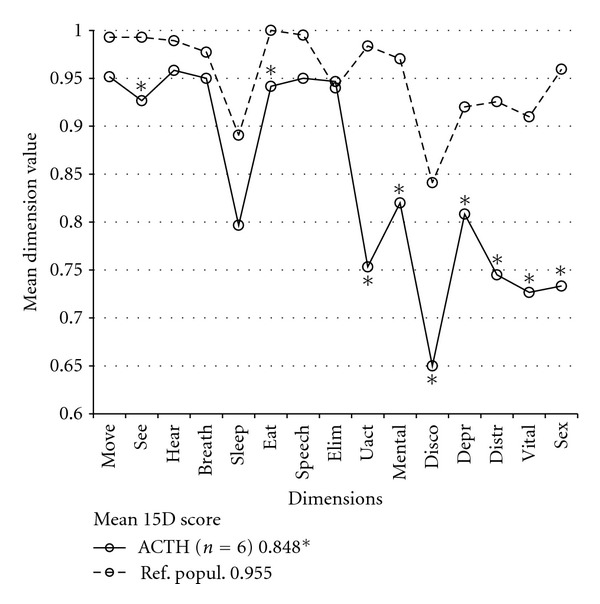
Mean 15D dimension values of Cushing syndrome patients (ACTH). The multiple statistically significant impairments in 15D dimensions are presented by an asterisk (*, *P* < 0.05) compared to the reference population (Ref. popul.). The mean 15D score of the ACTH group was significantly lower than that of the Ref. popul. (keybox). Abbreviations used: Move: moving, See: seeing, Hear: hearing, Breath: breathing, Sleep: sleeping, Eat: eating, Elim: eliminating, Uact: usual activities, Mental: mental functioning, Disco: discomfort, Depr: depression, Distr: Distress, Vital: vitality, Sex: sexual function.

The HRQoL of previously surgically treated PA patients was measured with the 15D instrument [13], a generic, 15-dimensional, standardized, self-administered questionnaire. For each of the 15 dimensions, the respondent chooses one out of five levels that best describes the state of health at the moment. A set of utility weights is used to generate (i) level values for each dimension on a 0-1 scale (1 = no problems on the dimension, 0 = being dead) and (ii) a 15D score representing overall HRQoL on a scale of 0-1 (1 = no problems on any dimension, i.e, “full” HRQoL, 0 = being dead). A 15-dimensional profile is drawn from the mean level values. A difference of ≥0.03 in the 15D score is considered clinically important. The dimensions of the 15D instrument and their abbreviations are presented in Figure 1.

All of the study patients made regular follow-up visits to endocrinologists at either Oulu University Hospital (OUH) or a general hospital in the OUH district, thus receiving medical therapy for possible pituitary hormone deficiencies (Table 1). However, the substitution of gonadal hormones was considered individually, and GH replacement therapy was not routinely used. Moreover, the patients were medically treated for hyperprolactinemia or active acromegaly, as clinically appropriate following the current international guidelines.

Demographic and PA-related data of the patients at the time of the inquiry, including hormonal status and possible pituitary hormone substitution or suppressive medication, were gathered from their medical and laboratory records in OUH. Hypopituitarism was defined as deficiency and substitution of at least one adenohypophyseal hormone (other than GH). PAs were classified by histological diagnosis, and gonadotropinomas were classified into the NF group. PAs 10 mm in maximum diameter were classified as macroadenomas. Acromegaly was considered to be controlled or uncontrolled, the latter including inadequately and poorly controlled disease, according to the consensus statements criteria [14]. Prolactinoma was considered to be controlled if serum prolactin was in the sex-specific normal range.

### 2.1. Ethics

The study protocol was approved by the Ethics Committee of the Northern Ostrobothnia Hospital District and all patients provided written informed consent.

### 2.2. Statistical Analysis

Quantitative data are presented as means, categorical data as percentages. The 15D profiles and scores of the study patients were compared with those of a representative sample of the general Finnish population [15], and the sample was weighted to correspond to the age distribution of each PA patient group. Sex-specific weighting of reference values was considered to be unnecessary since in the used age groups there were no statistical differences in 15D scores or dimensions values between genders. The differences between the mean 15D scores of the patient groups and their corresponding weighted reference populations, and between selected subgroups of the patients, were tested with the independent samples *t*-test. 95% confidence intervals (CIs) for proportional variables were determined based on normal approximation if *n* and *n* − *x* were >5; otherwise Wilson's method was used. Fisher's exact test was used for categorical variables. Both the independent samples *t*-test and multivariate linear regression analyses were performed to assess the independent association between current somatostatin analog (SSA, octreotide, or lanreotide acetate) or dopamine agonist (DA, bromocriptine, or cabergoline) treatment and the 15D scores and dimension level values of the patients. All the statistical methods except the CIs of proportional variables were carried out with PASW Statistics 18.0 for OsX software (SPSS Inc., Chicago, IL, USA). A *P* value < 0.05 was considered statistically significant. The CIs presented in Table 1 are not repeated in the text.

## 3. Results

In all, 109 out of 122 patients responded (89%). The mean time between the latest surgical intervention and the questionnaire was 6.3 years (range 1.0–13.5). Among the responders, five patients refused to participate. In addition, five patients could not fill in the 15D questionnaire due to mental disability (one congenital mental retardation, four acquired mental dementias). None of the acquired dementia patients had been subjected to transcranial surgery, multiple operations, or radiation therapy. The demographics, PA class or size distribution, or previous treatment history did not significantly differ between the respondents and nonrespondents. Since there was only one patient with a TSH-secreting adenoma, this case was excluded from the statistical analyses, leaving 98 study patients. Detailed data on demographics, clinical treatment, and the hormonal status of the study patients at the time of the 15D survey are presented in Table 1.

The majority (83%) of the PAs were macroadenomas, and 54% of the PAs were NF. Half (46%) of the patients were male, and the mean age at the time of inquiry was 53 years. Incidentally found PAs contributed 13% of all PAs, and 5% of the patients had pituitary apoplexy as the presenting symptom. Visual field defects were common (28% of all patients) prior to surgery; in most cases the deficits improved and only 7% had permanent deficiencies after treatment.

As a surgically treated PA was our primary inclusion criteria for this study, all patients had been operated at least once. At the time of the survey, all the study patients were surgically in stable condition, for example, there were no PA residuals distending the optic nerves. Most (92/98, 94%) of the patients were operated on transsphenoidally at least once. Twelve of the patients were operated on transcranially, and for six of them it was the only pituitary operation. The mean number of pituitary operations was 1.2 (range 1–4).

Patients with previous radiation therapy had hypopituitarism more often (12/14, 86%) than non-radiated patients (54%, *P* = 0.02). Transcranial surgery was also associated with hypopituitarism (11/12, 92%, *P* < 0.01). The number of patients receiving hormonal substitution therapy is presented in Table 1. Only 2 out of 50 (4%) hypopituitaric patients received GH substitution.

When all the PA patients were analyzed together (*n* = 98), there was a tendency for association between radiotherapy and impaired scores in the mental health (*P* = 0.09) or sexual function (*P* = 0.09) dimensions, but statistical significance was not reached. Medically treated adenohypophyseal hypopituitarism was common (*n* = 50, 51%), but it was not associated with impairment in any of the 15D dimensions. Patients' gender, treated diabetes insipidus (*n* = 6, 6%), and previous transcranial surgery (*n* = 12, 12%) were not associated with impairment in any of the 15D dimensions.

### 3.1. Analysis of PA Subclasses

#### 3.1.1. Cushing's Disease

At the time of the survey, five of all six CD patients were in complete remission and one had mild untreated hypercortisolism. One patient had panhypopituitarism. The HRQoL of CD patients was profoundly impaired; these patients had statistically significantly lower values than their reference population on nine of the 15 dimensions and in the 15D score (Figure 1). Due to the small number of CD patients, statistical subgroup analyses were impossible, but some details of this group are described here. All of the CD patients were operated on transsphenoidally once, while none were irradiated or operated on transcranially. One male and one female required adrenal extirpation, leading to mineralocorticoid and glucocorticoid substitution, the female also receiving androgen substitution.

#### 3.1.2. Acromegaly

**Figure 2:**
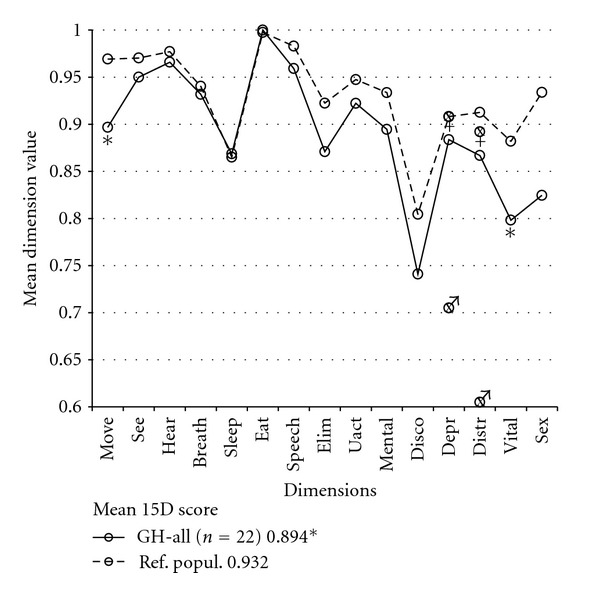
Mean 15D dimension values of acromegalic patients (GH-all). There was a statistical decrease in moving and vitality dimensions and the 15D score (keybox) compared to the reference population (Ref. popul.). Males (♂ symbol at the gender-specific mean of the particular dimension) had a significantly lower value in the depression and distress dimensions than did females (♀ symbol, resp.). 15D abbreviations are the same as in Figure 1. (*, *P* < 0.05).

At the time of the survey, nine of all 22 acromegalic patients (41%) were on SSA treatment (8 octreotide, 1 lanreotide), and two of the octreotide treated patients also received cabergoline (their PAs secreted both GH and PRL). Overall, acromegalics had significantly lower values than their reference population on the dimensions of moving and vitality and in the 15D score. In this acromegalic group, males had a higher rate of bodily pain as their subjectively stated symptom (8 complaints in 12 males) than did females (2 complaints in 10 females, *P* = 0.04). Furthermore, in the subgroup of acromegalics on SSA treatment, males showed more depression (*P* = 0.05) and distress (*P* = 0.02) than did the females (Figure 2).

**Figure 3:**
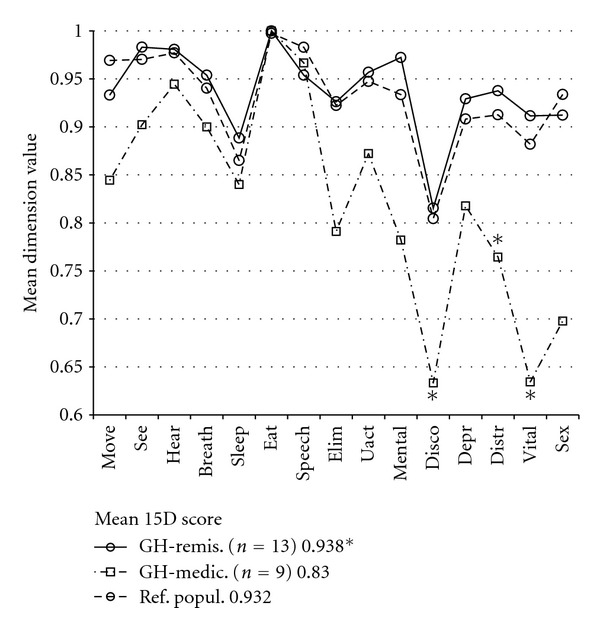
Mean 15D dimension values of acromegalic patients grouped by need for SSA treatment at the time of the questionnaire. When in remission (GH remis.), all the 15D dimension values were comparable to the reference population (Ref popul.). In contrast, acromegalics with SSA treatment (GH medic.) had significantly more discomfort and distress in addition to decreased vitality and the 15D score (keybox) compared to the Ref. popul. 15D abbreviations are the same as in Figure 1. (*, *P* < 0.05) acromegalics on SSA treatment versus Ref. popul.

Controlled and uncontrolled groups of acromegalics did not differ in mean 15D score or any the dimension. However, the acromegalics without SSA treatment (*n* = 13, including 9 controlled and 4 uncontrolled patients, Table 2) had the same HRQoL as their age-standardized reference population on all 15D dimensions (Figure 3). On the contrary, when satisfactory surgical remission of acromegaly was not reached and these patients were thus on SSA therapy (*n* = 9, including 6 controlled and 3 uncontrolled patients, Table 2), they had lower mean values than their reference population on the dimensions of discomfort, distress, and vitality and a lower 15D score. Furthermore, the mean hormonal outcomes (IGF-1 level and post-glucose nadir GH concentration) and controlled versus uncontrolled status (67% versus 69%, resp.) did not statistically differ between the SSA groups.

#### 3.1.3. Prolactinoma

**Figure 4:**
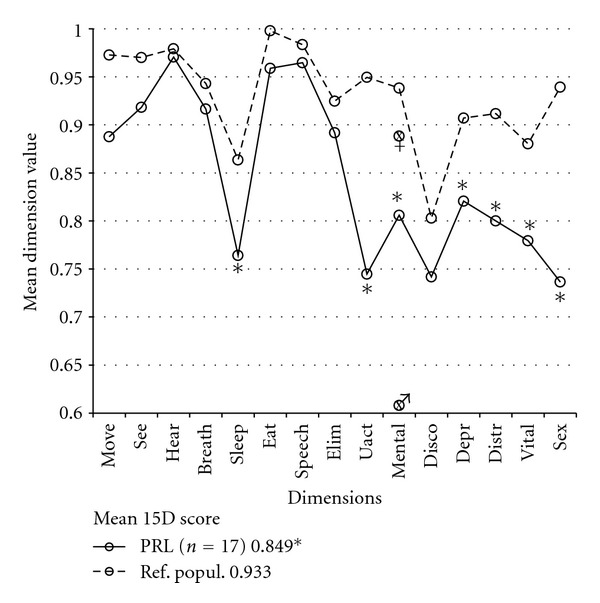
Mean 15D dimension values of prolactinoma patients (PRL). There was a statistical decrease in sleeping, usual activities, mental functioning, depression, vitality, and sexual function dimensions and in the 15D score (keybox) compared to the reference population (Ref. popul.). Males (♂, symbol at the gender-specific mean of mental dimension) had a significantly lower score in mental functioning than did females (♀, symbol resp.), but previous irradiation may be a confounding factor here (see text). The 15D abbreviations are the same as in Figure 1. (*, *P* < 0.05).

At the time of the survey seven of 17 (41%) of the prolactinoma patients were on DA treatment (4 bromocriptine, 3 cabergoline). Overall, the prolactinoma patients had multiple HRQoL impairments compared to their age-standardized reference population (Figure 4). There was no correlation between hyperprolactinemia (5 patients had a serum prolactin above normal range at the time of the survey) and mean 15D score or any of the dimension values. The prolactinoma patients with DA treatment had a lower value in the dimension of sexual activity (0.59 versus 0.84, *P* = 0.04) than the prolactinoma patients without DA, although the hyperprolactinemia profiles of these groups did not differ significantly at the time of the survey.

#### 3.1.4. Nonfunctioning Adenoma

There was no statistically significant difference between the patients with previously treated NF PA (*n* = 53) and their age-standardized controls in the mean level value of any of the 15D dimensions. Neither previous radiotherapy (*n* = 6, 11%) nor medically replaced hypopituitarism (*n* = 32, 60%) was associated with any decrease in the 15D mean dimension level values.

## 4. Discussion

The 15D HRQoL instrument was used in this PA study because reference values from a representative sample of the general Finnish population were available. In addition, in all important properties (reliability, content validity, discriminatory power, and responsiveness to change) the 15D has been found to be at least equal to other similar types of instruments [13, 16–18]. The 15D has also been used successfully in patients with acromegaly [19].

In the present study, the mean 15D score of previously surgically treated NF PA patients was comparable to that of the age-standardized general population, while the mean 15D score was significantly impaired in patients previously treated for hormonally active PA. Van der Klaauw et al. explored several HRQoL instruments in a comparison of different PA groups and found the greatest impairments in acromegalic and CD patients, while NF adenoma and prolactinoma patients showed the least impairments [10]. In a recent review, the HRQoL of CD patients seemed to be more impaired than that of other pituitary adenoma patients [2]. The present findings also showed that CD patients had the worst posttreatment HRQoL of all PA patients, as they had lower values on nine 15D dimensions (including all dimensions reflecting mental health) and in the 15D score. In contrast to van der Klaauw's findings, in the present study the acromegalic group as a whole had impaired values on only two 15D dimensions, while the prolactinoma patients had a lower values compared to their age-standardized population sample on six 15D dimensions. However, when taking into account the need of postoperative SSA medication, acromegalics without SSA treatment did not deviate significantly from their age-standardized reference population on any of the 15D dimensions (Figure 3) although the hormonal outcome in this group was equal to that of the SSA treated group (Table 2). In contrast, acromegalics with SSA treatment had a mean 15D score 0.10 lower than that of their controls, which is similar to the decrease in CD patients.

The HRQoL of prolactinoma patients has been found to be impaired in all of the SF-36 domains even when their prolactin levels were normal [20]. In the present study, the HRQoL of the patients with surgically treated prolactinoma was reduced on six 15D dimensions reflecting mainly psychic health. Interestingly, the prolactinoma patients with persistent hormonal excess requiring suppressive medical treatment did not have the aforementioned phenomenon of SSA-treated acromegalics. Despite treatment with DAs for residual hyperprolactinemia, these patients had impairment only on the dimension of sexual activity, while the mean level values on the other 15D dimensions and the 15D score were comparable with those of the prolactinoma patients without DA treatment. The reason for this impairment of sexual function cannot be determined with our data, as residual hyperprolactinemia was not associated with the 15D's sexual activity dimension.

Why was the HRQoL of acromegalics without SSA treatment as good as that of their age-standardized controls, even though active acromegaly causes for example, permanent skeletal changes? One of the severely impaired 15D dimensions (discomfort and symptoms) in the SSA-treated group is associated with bodily pain, while the others are associated with anxiety and exhaustion (Figure 3). There was no difference in age, sex, adenoma size, proportion having had transcranial surgery or radiation therapy, follow-up length, or hypopituitarism in acromegalics grouped by their need for SSA treatment. These facts could indicate that impaired HRQoL in the SSA treatment group was not caused by pretreatment skeletal changes, but rather by the activity of the acromegaly itself (i.e., indication for SSA) and the insufficiency of SSA treatment to completely normalize the GH-related metabolism of the patients, possible side-effects of SSA treatment, or discomfort and anxiety related to the need for regular SSA injections and/or surgically uncured acromegaly. Furthermore, the pharmacodynamics of SSA treatment may explain symptoms of extrahepatic acromegaly [4] despite the similar IGF-I profiles in the study's SSA groups. The present findings parallel those of Hua et al., who found impaired HRQoL parameters associated with lanreotide treatment in acromegalics whose disease was in a controlled state [5]. In addition, it is interesting to compare our results with those of a previous Finnish HRQoL study of treated acromegaly by Kauppinen-Mäkelin et al. [19], where no association was found between the GH-lowering medication and the 15D score. However, both SSA and DA users were analyzed as one group, and thus only 67% of the patients with GH-lowering medication received SSAs, compared with 100% in the present study. As DAs were not associated with impairment in the mean 15D score of the prolactinoma patients in the present study, the difference in GH-lowering medication might be an explanatory factor behind this difference. Moreover, there are interstudy differences in the proportion of patients in controlled status [14], in the present and the Kauppinen-Mäkelin studies (68% versus 51%, resp.) and in the use of SSAs (41% versus 21%, resp.). Furthermore, in the Kauppinen-Mäkelin study it was not stated whether the activity of acromegaly in patients with and without SSAs was similar. We cannot prove the causal association between SSA and impaired HRQoL due to our retrospective study design, but as there are no controlled prospective studies for HRQoL aspects of SSA treatments, the present results suggest the need for such studies.

Pituitary macroadenomas commonly cause hypopituitarism as a result of the mass effect of the tumor. Acquired hypopituitarism often persists after operation [21], and hypopituitaric patients usually need permanent substitution for their hormonal deficiencies. Even if the hormonal replacement therapy is formally appropriate, it does not necessarily reproduce normal plasma hormone profiles, and increased general and physical fatigue may be a sign of hypopituitarism in these patients [22]. Moreover, while deficiency of GH is one of the first deficits arising in hypopituitarism and HRQoL has been shown to improve during GH replacement therapy [23], the routine use of GH replacement is controversial. In the present study population, receiving aforementioned replacement therapies, medically treated hypopituitarism was not associated with lower HRQoL.

**Figure 5:**
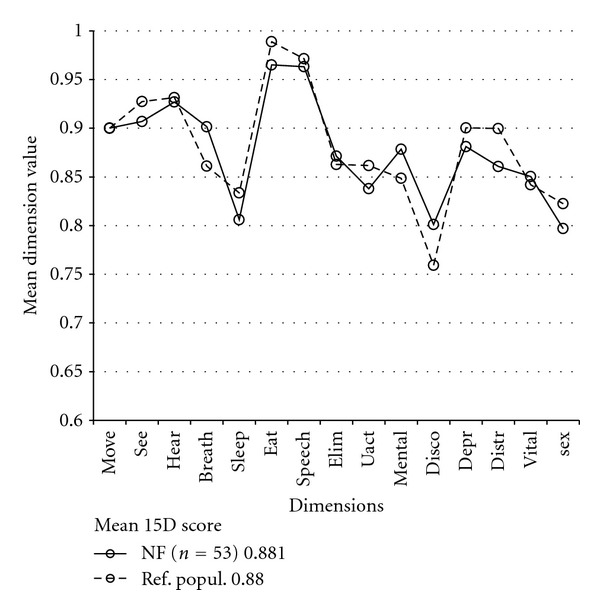
Mean 15D dimension values of NF adenoma patients (NF). There was no statistical difference in any of the 15D dimensions or in the 15D score (keybox) compared to the reference population (Ref. popul.). The 15D abbreviations are the same as in Figure 1.

In the studies by Nielsen et al. [11] and Page et al. [12] no statistically significant difference in HRQoL was found between operated NF adenoma patients and controls. On the contrary, in a study by Dekkers et al. [22] the HRQoL of operated NF patients was significantly reduced, as they reported increased mental and physical fatigue and reduced energy and motivation, but showed unaffected physical functioning. These symptoms may be caused by the aforementioned symptomatic hypopituitarism (93% of these patients were hypopituitaric). The present study is congruent with Nielsen and Page's results, as HRQoL was the same in the operated NF adenoma patients and their age-standardized reference population (Figure 5), despite the relatively high proportion (60%) of medically treated hypopituitaric patients in the study group. Moreover, of these patients, 11% had had radiotherapy, 23% multiple operations, 8% transcranial surgery, and 9% an episode of pituitary apoplexy.

The indications for operating on NF PAs vary. Even if a patient has no visual field defects or hypopituitarism, but an incidentally found adenoma shows a growth tendency, or if a patient on anticoagulant therapy has an incidentally detected macroadenoma, surgery may be indicated in an asymptomatic patient [24]. The present study confirms that overall morbidity after a transsphenoidal operation is minimal, and the HRQoL of these asymptomatic patients may be anticipated to remain the same after the operation.

There are some limitations in this retrospective study. First, we were not able to evaluate the possible HRQoL effect of given treatment, since we did not have pre-treatment 15D profiles and scores. Second, due to the low number of patients with CD and thyrotropinoma, we were not able to rank all the different PA classes according to their HRQoL impairment. Third, indications for medical therapy of post-surgically active acromegaly and hyperprolactinemia and rarely used GH-replacement therapy were not standardized. Lastly, due to the infrequency of some previously suspected negative predictors of decreased HRQoL of PA patients, especially transcranial surgery and radiotherapy, we cannot completely exclude their HRQoL effects.

## 5. Conclusions

Based on this retrospective study, the mean long-term HRQoL profile of patients with surgically treated NF adenoma seems to be comparable to that of an age-standardized reference population, while patients treated for ACTH- and PRL-secreting PAs seem to have significant HRQoL-impairments. In the acromegalic group of patients, we observed an association between SSA-treatment and decreased HRQoL, which seems to be an appropriate clinical topic for future prospective investigations. Medically treated adenohypophyseal hypopituitarism, transcranial surgery, and sellar radiotherapy did not seem to be associated with additionally impaired HRQoL.

## Figures and Tables

**Table 1:** The characteristics of the patient groups at the time of the 15D questionnaire. All patients were operated on at least once transsphenoidally. Percentages in all but the first row are proportions within the column. Mean follow-up time is the time from the latest operation. Hypopituitarism is deficiency of at least one adenohypophyseal hormone (other than GH). Multiple operations include possible pituitary re-operations or adrenal removal. Suppressive medications used are explained in the text. Hormonal status and medications reflect the situation at the time of the 15D questionnaire. NF: non-functioning, pts: patients, extirp.: extirpation, remiss.: remission, AHH: adenohypophyseal hormones, PA: pituitary adenoma, na: not applicable.

	ACTH			GH			PRL			NF			Total		
	*n *	value	95% CI	*n *	value	95% CI	*n *	value	95% CI	*n *	value	95% CI	*n *	value	95% CI
Number of patients (% of all pts)	6	0.06	(0.03–0.13)	22	0.22	(0.15–0.32)	17	0.17	(0.11–0.26)	53	0.54	(0.44–0.64)	98	1.00	
Demographics															
Mean age (yr)	6	34.8	(20.0–50.0)	22	45.0	(39.0–51.0)	17	46.4	(40.4–52.4)	53	60.0	(56.2–64.2)	98	52.8	(49.6–56.1)
Male sex (%)	1	0.17	(0.03–0.56)	12	0.55	(0.35–0.73)	5	0.29	(0.13–0.53)	27	0.51	(0.38–0.64)	45	0.46	(0.36–0.56)
Macroadenoma (%)	2	0.33	(0.10–0.70)	16	0.73	(0.52–0.87)	11	0.65	(0.41–0.83)	53	1.00	(0.93–1.00)	82	0.84	(0.79–0.90)
Apoplexy as a first symptom (%)	0	0.00	(0–0.39)	0	0.00	(0–0.15)	0	0.00	(0–0.18)	5	0.09	(0.04–0.2)	5	0.05	(0.02–0.11)
Visual defects pre-oper. (%)	0	0.00	(0–0.39)	2	0.09	(0.03–0.28)	4	0.24	(0.06–0.41)	21	0.40	(0.29–0.55)	27	0.28	(0.2–0.37)
Treatment															
Mean no. of PA operations	6	1.0	(1.0-1.0)	22	1.4	(1.1–1.7)	17	1.4	(0.9–1.2)	53	1.2	(1.1–1.4)	98	1.2	(1.1–1.3)
Craniotomy (%)	0	0.00	(0–0.39)	4	0.18	(0.07–0.39)	4	0.24	(0.06–0.41)	4	0.08	(0.04–0.2)	12	0.12	(0.07–0.2)
Multiple operations (%)	2	0.33	(0.19–0.81)	7	0.32	(0.16–0.53)	1	0.06	(0.01–0.27)	12	0.23	(0.13–0.36)	22	0.22	(0.16–0.33)
Radiation therapy (%)	0	0.00	(0–0.39)	6	0.27	(0.13–0.48)	2	0.12	(0.03–0.34)	6	0.11	(0.05–0.23)	14	0.14	(0.09–0.23)
Outcome															
Mean follow-up time (yr)	6	6.0	(1.1–10.8)	22	7.8	(6.1–9.5)	17	9.4	(7.5–11.2)	53	4.7	(3.6–5.7)	98	6.3	(5.4–7.1)
Hormonal cure by PA extirp. (%)	3	0.50	(0.1–0.7)	8	0.36	(0.2–0.57)	6	0.35	(0.17–0.59)	na	na	na	17	0.38	(0.23–0.5)
Suppressive medication (%)	0	0.00	(0–0.39)	9	0.41	(0.23–0.61)	7	0.41	(0.22–0.64)	na	na	na	18	0.18	(0.12–0.27)
Complete hormonal remiss. (%)	5	0.83	(0.44–0.97)	15	0.68	(0.47–0.84)	11	0.65	(0.36–0.78)	na	na	na	31	0.69	(0.22–0.4)
Hypopituitarism (%)	1	0.17	(0.03–0.56)	9	0.41	(0.23–0.61)	8	0.47	(0.26–0.69)	32	0.60	(0.47–0.72)	50	0.51	(0.41–0.61)
Mean no. of AHH's replaced	6	0.6	(–1.0–2.3)	22	1.0	(0.4–1.6)	17	1.0	(0.2–1.8)	53	1.2	(0.9–1.5)	98	1.1	(0.8–1.4)
Diabetes insipidus (%)	0	0.00	(0–0.39)	1	0.05	(0.01–0.22)	2	0.12	(0.03–0.34)	3	0.06	(0.02–0.15)	6	0.06	(0.03–0.13)
Visual defects post-oper. (%)	1	0.17	(0.03–0.56)	1	0.05	(0.01–0.22)	1	0.06	(0.01–0.27)	4	0.08	(0.03–0.18)	7	0.07	(0.04–0.15)

**Table 2:** Hormonal status of acromegalics in relation to SSA use at the time of the questionnaire.

Somatostatin analog treatment	Outcome according Giustina et al. [14]							Total
	Controlled		Inadequately controlled		Poor control	
No (59.1% of all 22)	9	69.2%	4	30.8%	0	0.0%	13	100%
Yes (40.9% of all 22)	6	66.7%	2	22.2%	1	11.1%	9	100%

Total	15	68.2%	6	27.3%	1	4.5%	22	100%
